# Fabrication of a GMA*-co-*EDMA Monolith in a 2.0 mm i.d. Polypropylene Housing

**DOI:** 10.3390/ma9040263

**Published:** 2016-03-31

**Authors:** Marcello Iacono, Damian Connolly, Andreas Heise

**Affiliations:** 1 School of Chemical Sciences, Dublin City University, Dublin 9, Ireland; marcello_iacono@hotmail.com; 2 Department of Science, Pharmaceutical and Molecular Biotechnology Research Centre (PMBRC), Waterford Institute of Technology, Cork Road, Waterford, Ireland; 3 Polymer Chemistry and Biopolymer Laboratory, Department of Pharmaceutical and Medicinal Chemistry, Royal College of Surgeons in Ireland, 123 St Stephens Green, Dublin 2, Ireland

**Keywords:** polymer monoliths, polypropylene housing, monolith anchoring

## Abstract

Polymers are interesting housing materials for the fabrication of inexpensive monolithic chromatography and solid phase extraction (SPE) devices. Challenges arise when polymeric monoliths are formed in non-conical, cylindrical tubes of larger diameter due to potential monolith detachment from the housing wall resulting in loss of separation performance and mechanical stability. Here, a two-step protocol is applied to ensure formation of robust homogeneous methacrylate monolith in polypropylene (PP) tubing with a diameter of 2.0 mm. Detailed Fourier-transform infrared (FTIR) spectroscopic analysis and Scanning Electron Microscopy (SEM) imaging confirm the successful pre-modification of the tubing wall with an anchoring layer of cross-linked ethylene dimethacrylate (EDMA). Subsequent formation of an EDMA-glycidyl methacrylate (GMA) monolith in the PP tube resulted in a homogeneous monolithic polymer with enhanced mechanical stability as compared to non-anchored monoliths.

## 1. Introduction

Over the past two decades, polymer monoliths have found many applications, broadly subdivided between chromatographic/electrophoretic separations and sample preparation in the form of solid phase extraction (SPE). A key challenge in these systems is to prevent monolith detachment from the housing wall due to the shrinkage during the monolith polymerization step, as that would negatively impact the separation performance and possibly the mechanical stability of the monolith. Generally, the risk of wall detachment increases with increasing housing diameter. A successful strategy is the covalent functionalization of the housing surface with polymerizable groups, e.g., vinyl groups in the case of acrylic monoliths as these can be incorporated into the monolithic network by stable covalent bond formation. In the vast majority of cases, monoliths are formed within fused silica capillaries (diameter < 300 µm) due to their facile covalent attachment to the wall via prior vinylization of the silica surface using reagents such as 3-(trimethoxysilyl)propyl methacrylate [1]. When a plastic housing, such as polypropylene (PP) or cyclic olefin copolymer (COC), is used then the surface attachment to the housing is somewhat more challenging due to the hydrophobicity and low reactivity of polyolefines. PP is particularly popular as a housing for polymer monoliths in SPE applications and formats have included pipette-tips [2,3,4,5,6,7,8,9,10,11,12,13,14,15,16,17,18,19,20] and syringe filters [21]. These monoliths have been used for the pre-concentration of peptides [2,8,10,11,15,16,18], proteins [5,20], fungicides [12], pesticides [3,4], plant flavones [7], pharmaceuticals [6,9,13,14,17] alkaloids [19], and metals [21] and fabrication protocols using pipette-tips have been reviewed by Blomberg [21] and Altun [22]. Surprisingly, few of these reports have addressed the issue of wall-attachment. Instead, the majority of workers have simply relied upon the monolith being held in position either due to swelling or by virtue of the fortuitous shape of the monolith (an inverted truncated cone) within its coned housing.

Fabrication of a polymer monolith within a fully cylindrical housing for separation applications is more challenging since the monolith cannot be mechanically confined at the narrower end of the mould. The grafting of acrylic oligomers onto the inner PP surface is challenging because, like all polyolefins, PP surfaces are hydrophobic and chemically inert under ordinary conditions. For these reasons, PP surfaces require chemical or physical treatment to allow further modification. Chemical modification of the interfacial polymer chains with polar groups such as hydroxyl, carbonyl, and carbonylic acid [23] by flame treatment [24], plasma treatment, [25,26], and low-pressure glow discharges [27], have been reported. However, all these methods require special equipment and are not routinely available in many research laboratories. Stachowiak *et al.* [28] have reported a two-step surface modification of PP micropipette tips and microfluidic channels containing abstractable hydrogens, which involves the initial UV-initiated immobilization of benzophenone derived free-radical initiator followed by the grafting of a polymer layer with a multiplicity of pendant double bonds from the surface prior to the subsequent polymerization of a monolith within the housing. The use of UV radiation and photo-initiators offers an excellent low cost alternative because of the simplicity and cleanness of this approach.

The aim of this study was to investigate whether robust homogeneous methacrylate monolith in PP tubing with a diameter of 2.0 mm can be produced, which to our knowledge is the largest diameter monolith formed to date in a UV-transparent polymeric housing. The challenge of the large diameter lies in the relatively larger effect shrinkage during the polymerization process has on the monolith dimension making it more prone to wall detachment. The results are reported in the form of a technical note that allows potential for commercial use. Special emphasis was given to the demonstration of the secure covalent attachment of the monolith to the tubing wall and the optimization of the initial inner wall functionalization to facilitate the same.

## 2. Results and Discussion

### 2.1. Surface Attachment of Monolith to PP

**Figure 1:**
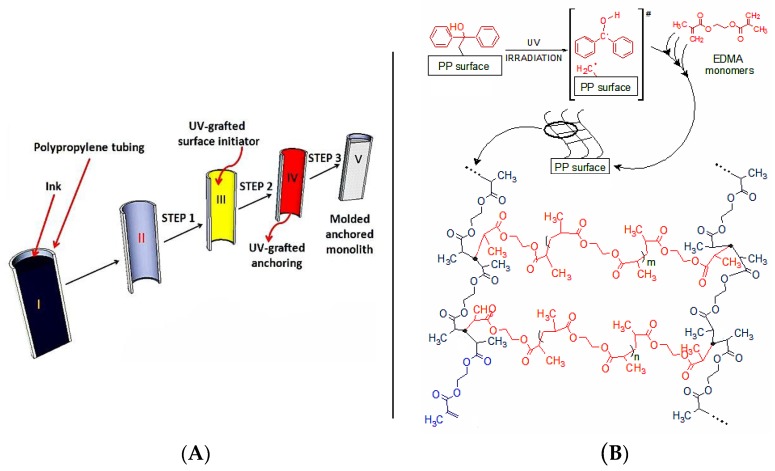
(**A**) Processing of an ink cartridge into an PP monolith device. The ink cartridge (I) was cleaned (II), followed by Step 1 (BP immobilization, III), Step 2 (grafting of p(EDMA), IV) and Step 3 (thermally-initiated monolith polymerization, V); (**B**) Schematic representation of the three-dimensional polymer network anchoring layer chemically bonded to the PP surface.

Conventional commodity PP tubing from ink pens was used as housing material. The device manufacture scheme is presented in Figure 1; after ink removal and tubing cleaning a three-step process was performed. The PP tubing ends were first fitted with plastic luer locks obtained from disposable steel needles which allowed watertight fluidic connection via syringe pump for flushing the tubing and the easy operation in an inert oxygen-free environment by plugging the housing fittings as appropriate. 

The PP housing used in the work has an inner volume of 3.14 mm^3^/mm. For the initial functionalization of the PP wall with acrylic monomers the two-step protocol proposed by Stachowiak *et al.* [28] was followed. It has to be noted that the original work by these authors was carried out on short (conical) micropipette tips of undisclosed diameter. In this work longer cartridges (up to 90 mm) were modified using the two-step process and then cut to the required length thereafter. The rationale for the Step 1 (Figure 1A) relies on the photochemistry of benzophenone (BP), which was first grafted onto the PP surface. This was achieved by filling the PP tube with a methanolic BP solution and irradiation with UV light under oxygen free conditions. Photo-chemically produced triplet states of the BP carbonyl group can abstract hydrogen atoms from almost all polymers, thus generating fast recombining semi-pinacolic radicals and surface-bound dormant radicals [29] to be regenerated in monolith formation step. Furthermore, the minimum concentration required to obtain complete surface coverage of the PP tube was approximately 0.002 wt % of BP in methanol [30] and so to ensure total surface coverage, a concentration of 5 wt % in methanol was used here. In this work the time between Step 1 (benzophenone immobilization) and Step 2 (ethylene dimethacrylate (EDMA) surface grafting) was kept to no longer than 24 h (the BP-PP surface is chemically stable for almost three weeks in dark conditions) [30]. Step 2 was performed using EDMA solutions in methanol wherein the surface radicals promoted a free radical polymerization of the EDMA diacrylate in solution and on the inner PP tubing surface under UV irradiation. This process resulted in an oligomeric cross-linked network chemically linked to the inner PP tubing cartridge surface (Step 2, Product B, Figure 1A). Key to this process is that Steps 1–2 were performed sequentially since a UV irradiation process with BP and EDMA present at the same time would promote the unwanted formation of BP-EDMA cross-products. Indeed, this problem is minimized by the two-step process because the semi-pinacolic radicals have a very short lifetime and preferentially recombine or terminate growing chains if the two processes are sequential [28]. This cross-linked surface polymer acted as an intermediate layer during the generation of the final monolith and ensured a uniform chemical adhesion of the EDMA*-co-*GMA monolith to the PP surface. 

### 2.2. ATR-IR Characterization of the Modified PP Surface

Attenuated total reflectance infrared spectroscopy (ATR-IR) is based on the interaction of an evanescent field penetrating the sample surface typically between 0.5 and 2 μm. For this reason, this technique is particularly suitable to monitor the success of the surface treatments of Steps 1 and 2 (see Figure 1A). Specifically it was possible to monitor the impact of UV treatments for different irradiation times during these steps. Therefore, for each of Step 1 and 2, after the surface treatment and subsequent washing/drying at room temperature with N_2_, the 90 mm PP tubing was cut into 5 mm sections and longitudinally cut open and flattened (5000 kg/cm^2^ during 5 min). In this way, a flat sample was formed with two different faces: one being the tubing inner surface (directly exposed to the BP/EDMA solutions) and the outer surface.

**Figure 2:**
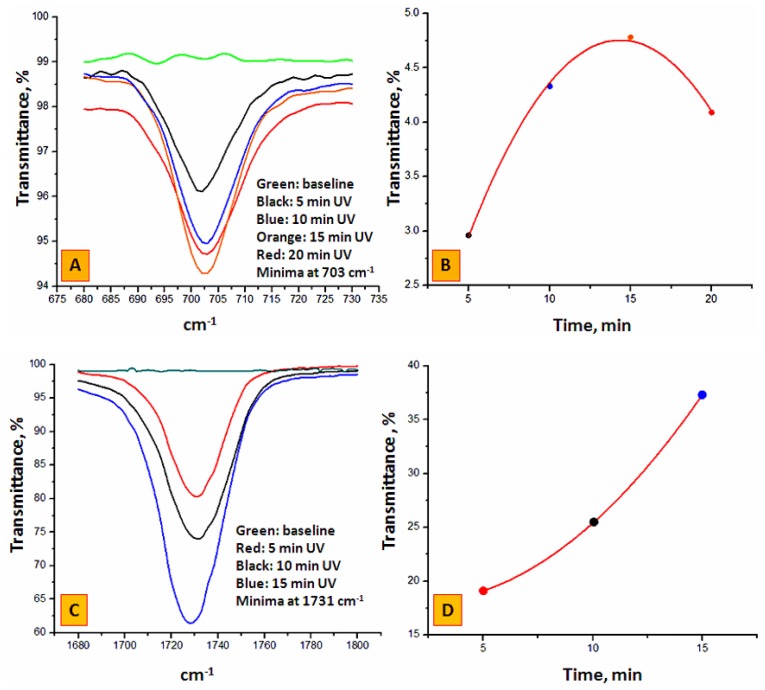
(**A**): ATR-IR spectra of the inner PP housing during BP immobilization after 5, 10, 15 and 20 min. UV exposure (see Step 1, Product A in Figure 1A); (**B**) Transmittance minima from (A), at 703 cm^−1^ as a function of UV irradiation time after baseline subtraction (see green line in (A)); (**C**) ATR-IR spectra of the inner PP surface during EDMA grafting (see Step 2, Product B in Figure 1A) using each time as starting material a 15 min. BP treated housing (see orange line in (A) and orange point in (B)); (**D**) Transmittance minima from (C) at 1731 cm^−1^ as a function of UV irradiation time after baseline subtraction (see green line in (C)). The polynomial best fit *y* = *a* + b* × time + c* × time^2^ is defined by the following coefficients: for Step 1, *a* = 0.505, *b* = 0.5918 min^−1^, *c*= −0.0206 min^−2^; adj. *R*^2^ = 0.996; for Step 2, *a* = 18.41, *b* = −0.423 min^−1^, *c* = 0.1122 min^−2^.

Figure 2A shows the ATR-IR signal at 703 cm^−1^ (corresponding to the immobilized benzophenone radical in their semi-pinacolic form) for the PP surface during Step 1 after UV light exposure for selected times. Each spectrum is the averaged accumulation of 1024 scans on the same sample. The spectra were identical for all 5 mm tubing sections obtained from three pieces of 90 mm tubing. The black, blue, orange and red lines are relative to four identical inner PP surfaces exposed to the UV radiation for 5, 10, 15 and 20 min, respectively, while the green line is the ATR-IR spectrum of an untreated PP surface exhibiting the same IR spectrum as the external PP surface irrespective of the UV exposure. In Figure 2B, the transmittance minima at 703 cm^−1^ (see colored points) are plotted using a polynomial best-fit curve (see the red curve) against time, after subtraction of the signal for the untreated sample. The first three experimental points (5, 10 and 15 min) in Figure 2B suggest that the BP concentration on the inner surface has progressively increased reaching a saturation plateau with increasing UV exposure time after which a decrease was observed, possibly due to the unwanted ingress of oxygen into the tubing over this prolonged period of time (>15 min). Therefore, an optimum irradiation time of 15 min was selected for further work. Figure 2C,D show three spectra collected for BP-modified PP samples after grafting EDMA for 5, 10 and 15 min. The band at 1731 cm^−1^ represents the carbonyl due to the grafted poly(EDMA) network. As expected, an increase of poly(EDMA) decoration with irradiation time (within the limits of a 15 min exposure) was observed due to the formation of a three-dimensional grafted polymer network. 

### 2.3. SEM Characterization and Stability of Polymer Monolith in the PP Housing

**Figure 3:**
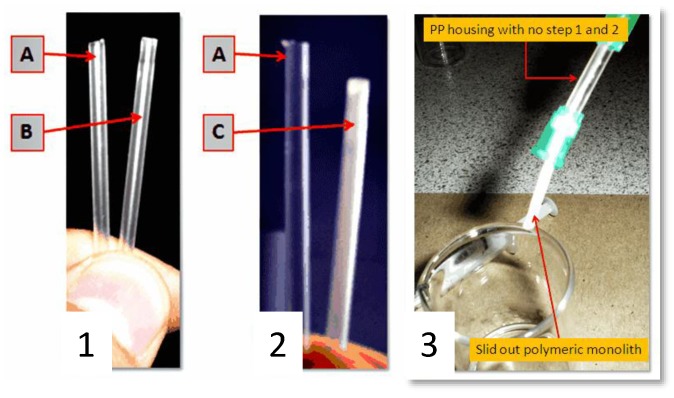
Image (**1**) shows the cleaned PP tubing A and the BP treated PP tubing B after the washing/drying step; Image (**2**) shows the cleaned BP treated PP tubing A and the EDMA treated PP tubing C after the washing/drying step. The inner surface of C shows a visible white layer; Image (**3**) shows a non-anchored monolith connected to a syringe pump under moderate pressure.

**Figure 4:**
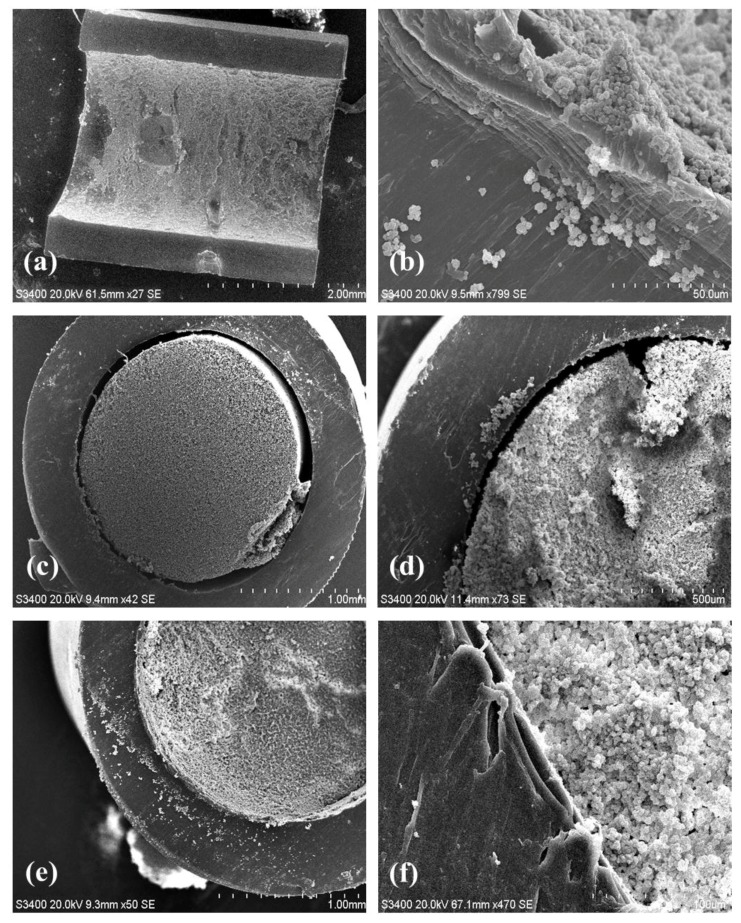
(**a**): Longitudinal sectioning of poly(EDMA) surface grafted tubing interior; (**b**): Grafted poly(EDMA) clusters at the wall boundary; (**c**,**d**): Polymer monolith formed within an unmodified PP tubing showing no wall attachment. (**e**,**f**): Secure attachment of polymer monolith to an p(EDMA)-grafted PP tubing. (magnification of 27× for (**a**); 799× for (**b**); 42× for (**c**); 73× for (**d**); 50× for (**e**); and 470× for (**f**)).

Additional proof of the anchoring system uniformity was obtained by simple visual examination of the tubing and by scanning electron microscopy. Step 1 did not change the physical appearance of the transparent PP tubing whereas Step 2 resulted in a readily observable dense white skin bonded to the inner surface (Figure 3). The residual white solution that was flushed from the tubing after Step 2 was opaque with macroscopic poly(EDMA) granules in suspension resulting from unbound polymer formed by the free radical polymerization. Figure 4a shows a SEM image of the longitudinally sectioned tubing which clearly shows the presence of plaques of grafted polymer on the inner surface, particularly noticeable against the ungrafted cut faces of the tubing. Figure 4b is a magnified close up showing the boundary between ungrafted PP (characterized by parallel striations due to the PP manufacturing process on the bottom left) and the grafted clusters of poly(EDMA). 

The subsequent formation of a p(GMA)*-co-*(EDMA) monolith within the confines of this tubing was carried out both in unmodified tubing and in EDMA-modified tubing. In the latter case, three phenomena were expected to contribute to the stabilization of the monolith in the tubing: (1) Interpenetration of the p(EDMA)-*co*-(GMA) networked with the already present p(EDMA) anchored network; (2) frictional forces between the p(EDMA) anchoring layer and the porous monolith; (3) chemical binding with the unreacted EDMA double bonds of the anchoring layer and covalent incorporation into the GMA-*co*-EDMA network. While analytically difficult to verify, the presence of statistically unreacted double bonds in the anchoring layer is reasonable to assume based on the fact that double bond conversion in network formation is never quantitative.

Clearly the monolith formed in the unmodified (and without the chemically linked p(EDMA) anchoring system) tubing (Figure 4c,d) was not bound to the wall and a large void around the perimeter of the monolith is clearly visible. Indeed, this monolith could be easily extruded from the tubing by the application of modest pressure from a syringe pump during subsequent washing steps (Figure 3). Conversely, the monolith formed in the EDMA-modified tubing was intimately attached to the tubing surface as shown in Figure 4e,f. The monolith had a globular structure typical of poly(GMA*-co-*EDMA) monoliths (Figure 4f) and there was no optical evidence of an unwanted radial gradient of monolith density from the wall through the center of the monolith as shown in Figure 4c,e. Such a gradient has previously been reported by Nesterenko *et al.* [31] who formed a monolith within 0.8 mm i.d. titanium tubing and observed significant differences in monolith density (pore size) across the monolith radius, which could only be mitigated by the use of a complex thermal gradient during monolith polymerization. Presumably the very different thermal properties of PP relative to titanium are responsible for this observation. Indeed, titanium has a good thermal conductivity. For this reason, the first nuclei of the p(EDMA)-*co*-(GMA) monolith, whose formation is ruled by the thermal initiation, will presumably form faster close to the metallic housing inner walls. On the contrary, polypropylene has a poor thermal conductivity which might promote more uniform heating and formation of p(GMA)-*co*-(EDMA) nuclei. 

All monoliths in the 2 mm PP housing were extensively solvent flushed by connecting them to a syringe pump using two Luer Lock connectors. Over a period of 8 hours the devices were flushed with methanol at a maximum flow rate of 500 µL/min. This corresponds to a pressure at the device inlet of 5.546 × 10^−4^ Pa (*p* = surface × linear force of syringe pump). During this time no change in their morphology was observed. In order to further investigate the stability of the monolith in the housing an approach was applied whereby the pores were deliberately blocked. To achieve that, a slurry of commercial silica gel (35–75 μm) was pushed through the monolith by a syringe pump. The blocked 15 mm long device withstood the maximum force of the syringe pump (pressure of 5.546 × 10^−4^ Pa) over the test period of up to 8 h. The tests were repeated with shorter segments of the devices but only for segment lengths below 6 mm the monolith could be extruded under these conditions. These results were reproducible over several batches. Note that the monolith without the anchoring layer could be extruded at moderate pressure from a syringe pump. 

## 3. Materials and Methods 

### 3.1. Materials 

Benzophenone (ReagentPlus^®^, 99%, Sigma Aldrich, Tallaght, Ireland), azobisisobutyronitrile (AIBN, 98%, Sigma Aldrich, Tallaght, Ireland), ethylene dimethacrylate (EDMA, 98%, contains 90–110 ppm monomethyl ether hydroquinone as inhibitor, Sigma Aldrich, Tallaght, Ireland), glycidyl methacrylate (GMA, 97%, contains 100 ppm monomethyl ether hydroquinone as inhibitor, Sigma Aldrich, Tallaght, Ireland), 1,4-butanediol (ReagentPlus^®^, 99%, Sigma Aldrich, Tallaght, Ireland), 1-propanol (ACS reagent, ≥99.5%, Company, City, Country), methanol (MeOH, anhydrous, 99.8%, Company, City, Country), acetone (anhydrous, 99.8%, Sigma Aldrich, Tallaght, Ireland), ethanol (EtOH, anhydrous, 99.8%, Company, City, Country) and AtmosBags© were purchased from Sigma Aldrich (Sigma Aldrich, Tallaght, Ireland). All materials were used as received. Deionised water was produced with a Millipore Direct-Q5 (Millipore, Bedford, MA, USA). Polypropylene tubing (2 mm i.d., up to 90 mm long) was removed from Crystal BIC^©^ pens (Société Bic, Clichy, Hauts-de-Seine, France). Luer-lock polypropylene syringes (1 mL) were provided by Lennox Supplies Ltd. (Lennox Supplies Ltd., Dublin, Ireland). Silica gel, technical grade, pore size 60 Å, 200–425 mesh particle size 35–75 μm was obtained from Sigma Aldrich.

### 3.2. Instrumentation

A Perkin-Elmer Spectrum 100 (Perkin-Elmer, Dublin, Ireland) was used for collecting attenuated total reflectance Fourier transform infrared (ATR-FTIR) spectra in the spectral region of 650–4000 cm^−1^. A Hitachi S-3400N scanning electron microscope (Hitachi, Maidenhead, UK) with Image J software (Image J 1.48v, National Institute of Health, Bethesda, MD, USA) was used for monolith characterization and all samples were previously gold sputtered using a Quorum Technologies 750T sputter coater (Quorum Technologies, Sussex, UK). A Memmert UN750 laboratory oven (Lennox Supplies Ltd., Dublin, Ireland) was used for drying and polymerization purposes. The balance used was a Sartorius Extend (Sartorius, Goettingen, Germany) and a Harvard Apparatus PHD2000 syringe pump (Harvard Apparatus, Holliston, MA, USA) was used for flushing polypropylene tubing and tubing-bound polymer monoliths. UV irradiation was achieved using a Fusion UV LC-6B Curing station, equipped with a 360–370 nm UV lamp (300 watt/inch mercury H-bulb) and a speed-controlled moving conveyor belt (Heraeus Noblelight Fusion UV Inc., Gaithersburg, MA, USA).

### 3.3. Methods 

#### 3.3.1. Preparation of Polypropylene Housings

Polypropylene housings were drained of ink and washed sequentially with acetone and ethanol before drying in an oven at 40 °C for 60 min (for a 90 mm PP tubing *ca.* 100 mL of each solvent was used in 3, 4 aliquots during 60 min). Pre-cut lengths of tubing were then fitted at each end with female luer lock connectors cut from disposable hypodermic needles. The water-tight connections were further sealed with Teflon tape while avoiding the potential for unwanted screening of incident UV radiation due to poor positioning of the tape. Moreover, the luer lock-polypropylene tubing geometry assured the minimum dead volume within the device.

#### 3.3.2. Immobilization of Benzophenone and EDMA

A solution of 5 wt. %. benzophenone in MeOH was deoxygenated for 15 min by bubbling N_2_ gas through the solution. Polypropylene tubes were carefully filled with the solution using 1 mL plastic syringes inside a N_2_-filled AtmosBag^©^. Visual inspection before UV irradiation confirmed the absence of gas bubbles confined inside the sealed housing or leaks as possible sources of in homogeneities in the anchoring system. The filled tubes were then treated with a Fusion UV lamp for a total of 15 min. The moving conveyor belt passed the tubes beneath the lamp several times, and the tubes were re-positioned after each pass to ensure uniformity of irradiation; the integrated air-cooling was used to minimise increases in temperatures significantly above ambient. The tubing was then drained, washed with MeOH and dried with a flow of N_2_. Benzophenone-modified tubing was stored in the dark and used within 24 h. Before subsequent modification with EDMA, the luer lock connections (also being constructed of polypropylene) were exchanged. The EDMA immobilization procedure was the same as for benzophenone, using deoxygenated 5 wt. % EDMA in MeOH.

#### 3.3.3. Polymerization of GMA-co-EDMA Monoliths in Polypropylene Housings

A mixture of GMA (225 mg, 1.58 × 10^−3^ mol), EDMA (75 mg, 3.78 × 10^−4^ mol), 1-4 butanediol (280 mg, 3.11 × 10^−3^ mol), 1-propanol (350 mg, 5.82 × 10^−3^ mol), water (70 mg, 3.88 × 10^−3^ mol) and AIBN (3 mg, 1.83 × 10^−5^ mol) was deoxygenated by a N_2_ stream for 15 min and filled into the EDMA-modified polypropylene housings. The housings were positioned vertically in an oven at 70 °C for 22 h and then, once at room temperature, connected to a syringe pump by Luer Lock connectors and washed with MeOH to remove porogens and un-reacted monomers at 500 µL/min. The final devices were stored at room temperature in sealed boxes to prevent dust and/or chemical contaminations. In Table 1 the main physical dimensions of the system are listed.

#### 3.3.4. Stability Test

Two milliliters of silica gel (particle size 35–75 μm) was mixed with 3.0 mL of distilled water to make a silica gel slurry. A 1 mL Luer Lock Manual Syringe was half filled with the slurry and connected to a pre-flushed (20 mL of water at low flow rate) 15 mm long PP monolith and attached to a syringe pump. The syringe pump was turned on at 100% force (5.546 × 10^−4^ Pa). Clogging of the monolith by the silica gel slurry was evident within 1 min. The pressure was maintained for 8 hours with no visible change to the monolith. The same test was repeated with 6 mm PP devices and immediate extrusion of the monolith was observed. 

## Figures and Tables

**Table 1:** Physical dimensions of the monolith in the PP housing.

PP Monolith	Dimension
Outer housing diameter	3.0 × 10^−3^ m
Inner housing diameter	2.0 × 10^−3^ m
Monolith mass per tool length	0.920 g/m
Monolith surface per tool length	480 m^2^/m ^a^
Inlet or outlet area of the monolith	3.14 × 10^−6^ m^2^
Mass of anchoring system per tool length	0.019 g/m
Epoxy group content	4.81 ×10^−3^ mol/m ^b^

^a^ Value is derived from literature data [32] and the known mass per length; ^b^ This value is based on the assumption that all the GMA monomers used to cast the monolith are present in the final monolith.
